# Social and environmental predictors of walking among older adults

**DOI:** 10.1186/s12877-016-0327-x

**Published:** 2016-08-23

**Authors:** Marcia G. Ory, Samuel D. Towne, Jaewoong Won, Samuel N. Forjuoh, Chanam Lee

**Affiliations:** 1 Department of Health Promotion and Community Health Sciences, Texas A&M Health Science Center School of Public Health, College Station, Texas, 77843 USA; 2 Department of Landscape Architecture and Urban Planning, Texas A&M, College Station, Texas, USA; 3 Department of Family & Community Medicine, Texas A&M Health Science Center, College Station, Texas, USA; 4 Baylor Scott & White Health, Temple, Texas, USA

**Keywords:** Older adults, Walking, Social environment, Physical activity, Healthy aging, Health and place

## Abstract

**Background:**

Regular physical activity (PA) is a major factor in maintaining health in aging populations. This study examines the influences of sociodemographic, health, and environmental characteristics on older adults’ walking behaviors, and the role physicians can play in promoting physical activity.

**Methods:**

Online and paper surveys (*n* = 272) were distributed to community-dwelling older (age ≥ 60) adults from a large integrated healthcare system in two counties in Central Texas. Descriptive statistics were utilized to characterize participant’s walking behaviors and places. Multivariate logistic regression was employed to predict being: 1) a frequent walker (i.e., walking at least three times a week); and 2) meeting the Centers for Disease Control and Prevention (CDC) PA recommendation through walking (i.e., walking ≥150 min per week), while considering sociodemographic, health, and environmental factors.

**Results:**

Individuals had a median age of 69 years, were of both genders (50.37 % female), and were primarily non-Hispanic White (84.87 %). While the majority (59.55 %) walked at least three times a week, only 27.86 % walked ≥150 min a week. Factors associated with a lower likelihood of being frequent walkers included experiencing poor mental health in the past month (OR = 0.345, 95 % CI = 0.185–0.645) and residing in areas with low or moderate (versus high) perceived neighborhood cohesion (OR = 0.471, 95 % CI = 0.228–0.974), while those in Census Tracts reflecting populations with a lower median age were more likely to report frequent walking behavior (OR = 1.799, 95 % CI = 1.034–3.131). Factors associated with a lower likelihood of meeting the CDC PA recommendation included being 60–69 years (versus 70 years or older) (OR = 0.538, 95 % CI = 0.290–0.997), experiencing poor mental health in the past month (OR = 0.432, 95 % CI = 0.198–0.944), and lacking social support for walking (OR = 0.383, 95 % CI = 0.154–0.957).

**Conclusion:**

Given the health benefits, PA promotion must be seen as a national responsibility. In particular, physicians have a major role to play in communicating the importance of PA to their older patients and making discussions about strategies for overcoming barriers to walking an integral part of their clinical encounter with these patients.

## Background

There is an emergent awareness that health benefits derived from physical activity (PA) do not diminish over the life-course, and that PA is also important for older adults as a way of both preventing and managing chronic diseases and disabilities [1, 2]. Additionally, there is a growing literature on the impact of the built environment on the amount and types of PA, with environmental design features promoting or hindering activity patterns [3–6]. Thus, the identification of places where older adults engage in PA and the factors influencing the amount of PA is critical for promoting population health [7].

An evolution in the types and settings of PA being studied has occurred over the past decades. Earlier clinical perspectives focused on intensive exercise regimens for cardiovascular benefit that typically required exercise equipment in a gym setting [8]. In contrast, the current public health perspective stresses that PA should be incorporated into everyday routines and settings such as people’s neighborhoods [9]. Additionally, physicians are seen as playing a critical role in assessing current PA levels and advising their patients on strategies for being more active [10].

Yet, national statistics indicate that while the majority of older adults engage in some minimal PA (e.g., walking up to 3 days a week), the majority fail to meet the recommended PA guidelines for engaging in PA (i.e., at least 150 min of moderate physical activity per week) [11]. This has resulted in a growing interest in understanding linkages between health and place, and how different dimensions of the environment interact with lifestyle factors to influence a variety of health outcomes across the life-course [7, 12, 13].

While there are a myriad of ways to be physically active, walking is one of the most popular forms of PA, especially among older adults [14]. Walking behaviors resonate with environmental studies attempting to better understand where, why, and how community members engage in PA [15, 16]. Studies of older adults typically examine the amount of PA reported as well as barriers and facilitators to exercise and clinical or behavioral intervention effects [2, 17]. What is less known is how the physical and social environments interact with socio-demographic and self-assessed health statuses to predict who is most sedentary or most active [18].

Our study aims are to ascertain both individual and environmental factors associated with two threshold PA patterns among older adults including: 1) being a frequent walker (i.e., walking at least three times a week); and 2) meeting the Centers for Disease Control and Prevention (CDC) PA recommendation (i.e., at least 150 min of moderate PA per week) [14] through walking. A novel aspect of this study is the examination of different features of the social and physical environments and the assessment of the similarities and differences in factors related to meeting either minimal PA levels or engaging at higher levels in line with meeting the recommended PA guidelines.

## Methods

### Target population and setting

The target population includes community-dwelling older adults (age ≥ 60) drawn from a large integrated healthcare system in two counties (Bell and Brazos) in Central Texas. This analysis is part of a larger study that examined environmental safety factors associated with various health behaviors and health outcomes among adults 50 years or older. Four cities (Temple and Killeen from Bell County, Bryan and College Station from Brazos County) were chosen to provide adequate subject samples and environmental variability for this study. The counties were peri-urban with no large metropolitan cities, and represent understudied environmental settings in walkability research. With our interest in understanding factors associated with walking behaviors in older adults, persons 60 years or older were selected as the population group of interest for this analysis. We chose this age because this is the age when older adults are eligible for Older American Act benefits for health promotion and disease prevention [19].

### Recruitment and data collection techniques

Electronic medical records of patients who lived in our study areas were utilized to conduct initial subject screening by age and geographic residence. To target active individuals, only those that had at least one clinical encounter in the Family Medicine Department during the past 3 years were eligible to be included in our sampling frame. From the selected list, individual names were randomly drawn, reviewed, and approved by their primary care physician. Approximately 1000 letters were mailed out every month except during the winter months when people are less likely to walk outside. We further restricted our study sample to those who: a) did not have any difficulty to read, write, and speak English; b) were able to walk at least three city blocks (or for about five minutes); c) were not terminally ill; and d) did not live in a nursing home or assisted living community. In order to ensure the individual’s capability of walking or engaging in PA, screening questions for the ability to walk were included at the beginning of the survey instrument.

Participants were given the option to choose whether they wanted online (69.5 %) or paper surveys (30.5 %). The survey took approximately 20 min to complete, and included questions on demographic, health, and environmental factors potentially related to walking. A $10 gift card was offered at the completion of the full survey. Further, power analyses were conducted prior to recruitment indicating a sample of at least 250 was needed to detect differences (80 % power) in major outcomes. Assuming 60 % as those meeting the PA recommendation and 40 % as those not meeting the recommendation [20], a total of 250 patients can achieve 80 % power to detect a difference of 0.36 standard deviation with a significance level (alpha) of 0.05 using a two-sided, two-sample *t*-test. This metric was exceeded in the current analysis.

Ethical approval was sought and obtained from the institutional review board or IRB (ethics committee) of Baylor Scott & White Health before the study began. Written informed consent was gained from participants via online surveys or paper surveys. Consent was recorded/documented via online through Qualtrics secured survey management and via in-person (paper) retrieved from participants by research staff and kept in a secure file to be destroyed at the end if the study. This consent procedure was approved by the Baylor Scott & White Health IRB.

### Outcomes of interest

Recent literature differentiates different types of walking (e.g., walking for transportation and walking for recreation). Walking for “any purpose” was viewed as the most appropriate construct for older adults whose days may not be as structured around work or recreation as younger persons. To ascertain *frequency of walking behavior*, we defined PA based on the number of days an individual walked during a ‘typical’ week. We used the question: ‘How many days in a typical week do you walk in your neighborhood for any purposes?’ for this measure. Based on prior literature [21–23], we created a variable to assess whether an individual participated in walking for any purposes for less than 3 days during a typical week (defined as non-frequent walking) versus 3 or more days (defined as frequent walking).

To ascertain whether patients were meeting the Surgeon General’s recommendations through walking, we assessed the average minutes of PA during a typical week. To evaluate whether individuals were meeting the recommended level of PA set by the CDC (i.e., 150 min of moderate activity) [14], we used a combination of the self-reported average number of minutes of PA through walking per day and the average number of days an individual participated in PA through walking per week in one’s neighborhood. The result was the average weekly minutes of PA through walking. Previous evidence suggests that walking at a normal pace may be equivalent to moderate intensity PA [24]. Therefore, walking will likely capture at least moderate PA with regard to the national guidelines.

### Individual-level variables

As noted below, the survey items employed came from previously validated survey instruments (i.e., the Behavioral Risk Factor Surveillance System or BRFSS [25, 26] and the Neighborhood Environment Walkability Scale or NEWS [27, 28]), or reflect an assessment battery used by our study team in its health and environmental studies. Although the study targeted those 60 years or older, we wanted to examine differences among this group. Age was coded as 60–69 years (younger) and 70 years or older (older). Education was coded as having a high school education or less versus higher. Gender was coded as male or female. Race/Ethnicity was coded as White-non Hispanic or minority individuals (e.g., encompassing African Americans, Hispanic, and other racial groupings).

Health status was measured using two standard survey items assessing physical and mental well-being from the CDC health-related quality of life assessment battery used in the Behavioral Risk Factor Surveillance System or BRFSS [25, 29]. The following was used to assess physical well-being: ‘Now thinking about your physical health, which includes physical illness and injury, for how many days during the past 30 days was your physical health NOT good?’ The following was used to assess mental well-being: ‘Now thinking about your mental health, which includes stress, depression, and problems with emotions, for how many days during the past 30 days was your mental health NOT good?’ We wanted to determine differences in those with high versus low number of days in either physical or mental well-being (separately). Therefore, we dichotomized these variables into high (at or above the upper quartile) versus low (less than the upper quartile), which resulted in a split of less than 2 days versus greater than or equal to 2 days in the past 30 days for both physical and mental well-being in two separate variables.

### Social support variables

Marital status (never married or divorced, widowed or separated versus married or living with a partner) was included to assess differences in social support influenced by having a spouse/partner. We also included items to tap social engagement for walking [30]. We examined whether individuals reported having someone to walk with to assess human social support for walking. We also included an item to reflect human-animal companionship as a potential motivator for walking. A new combined measure of social support was constructed using the questions: *‘Is there someone in your household you go walking with?’* and *‘Is there a dog in your household that you usually walk?’* Social support for walking was coded on a scale of 0 to 2, with 0 representing a negative (no) response to both items, 1 representing a single affirmative (yes) response, and 2 representing affirmative responses to both items.

### Environmental variables

In addition to individual respondent’s age, we wanted to reflect median neighborhood age. Median age at the participant’s Census Tract was included to identify differences across younger versus older neighborhood populations. This was coded as at or below the median versus higher than the median among those Census Tracts that were included in the study population.

We also included respondents’ perception of their neighborhood, measured with the Neighborhood Environment Walkability Survey (NEWS) instrument [27] which included multiple domains of variables capturing neighborhood features influencing walking. Neighborhood traffic safety was included to account for variation in perceived safety. This was coded as a scaled variable with the following 4 items: 1) There is so much traffic along the street I live on that it makes it difficult or unpleasant to walk in my neighborhood; 2) There is so much traffic along nearby streets that it makes it difficult or unpleasant to walk in my neighborhood; 3) There are sidewalks or protected walkways (e.g., trails) in my neighborhood; and 4) There are crosswalks and pedestrian signals to help walkers cross busy streets in my neighborhood. All questions were on a scale from 1 to 4, with 4 representing all responses indicative of the poorest safety and 16 indicative of the best safety. To preserve cell size, we combined this scale into at or below the lower quartile or low (4–10), moderate (11, 12), and at or above the upper quartile or high (13–16) according to quartile splits. Perceived neighborhood fall injury risk was also included and assessed on a single item: “*I am worried about falling when I walk in my neighborhood*.” The scores on a scale ranging from 1 (strongly disagree) to 4 (strongly agree) and were dichotomized into agree or disagree.

Finally, neighborhood cohesion was captured by combining responses from the following 5 questions: 1) I see and speak to other people when I am walking in my neighborhood; 2) Many people walk or bike in my neighborhood; 3) The streets and walkways in my neighborhood are clean and well maintained; 4) Walkers and bikers on the streets in my neighborhood can be easily seen by people from their homes; and 5) My neighbors could be counted on to help in case of need. All questions were on a scale of 1–4, with 5 representing all responses ‘strongly disagree,’ with a maximum of 20 representing all responses ‘strongly agree.’ To preserve cell size we combined this into at or below the lower quartile or low (5–14), moderate (15–17), and at or above the upper quartile or high (18–20) according to quartile splits. Similar variables have been used to describe neighborhood cohesion elsewhere [3, 16].

Information on where individuals were physically active was also included for descriptive analyses. The following item was used: *When you walk in your neighborhood, where do you walk at least once a week? (Check ALL that apply)*. This was coded as green areas (parks or trails/paths in the park, trails or paths not in the park, natural green spaces or near water features (e.g., forests, lakes); neighborhood streets; gyms or fitness centers; schools or tracks in the school; malls or shopping centers (e.g., Wal-Mart, HEB); and other.

### Statistical analyses

SAS 9.4 was used to run all analyses. Descriptive analyses were utilized to characterize the population and walking patterns. Chi square tests were used to test whether there were significant differences between groups across our outcomes of interest. Multivariate logistic regression was used to assess the likelihood of being frequent walkers vs. non-frequent walkers and meeting or failing to meet the Surgeon General’s PA recommendation. Odds ratios and 95 % upper and lower confidence intervals are reported.

### Survey responses

**Fig. 1:**
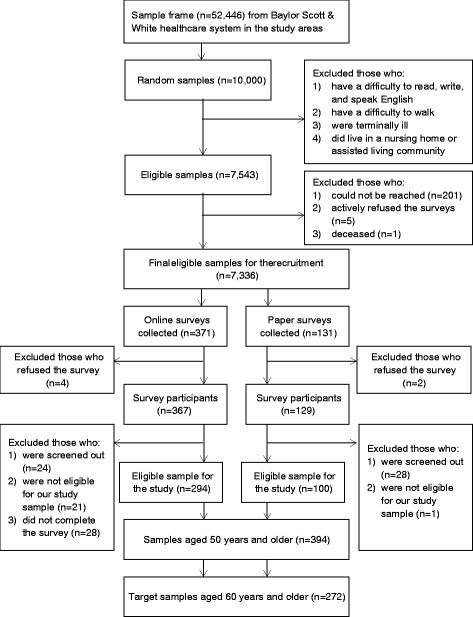
Flow chart of survey response and data collection

Given the approximately 52,000 individuals (age ≥ 50) in the sample frame, we randomly sampled 10,000 subjects, who lived in our study areas and were seen in primary care (Family Medicine) clinics during the past 3 years. After primary care physicians identified potentially eligible subjects on the sampling lists, a total of 7336 recruitment letters were sent out from October 2013 to June 2014. A total of 496 individuals participated in the surveys. Of the 496, 80 participants who did not meet the study screening criteria and did not complete the survey were excluded. Based on our expanded eligibility criteria (e.g., living in our study areas, not living in assisted facilities), 22 of the remaining 416 were excluded. Of these 394, 122 younger than 60 years were excluded to focus on our target population group (60 or older). Analyses for this study are based on the remaining 272 subjects. Figure 1 illustrates the flow of survey response and data collection.

## Results

### Participant characteristics

As indicated in Table 1, the median age of the study participants was 69 years (range: 60 to 92). They were of both genders (50.37 % female), primarily white-non Hispanic (84.87 %), educated (76.01 %) having more than a high school degree), and married (77.04 %). They generally reported good health, with the majority reporting relatively few days (≤1 day per month) with poor physical (73.80 %) or mental health (73.53 %). While only 19.85 % had both human and animal companions to walk with, almost half (49.26) had a least one source of social support for walking. Very few participants expressed worry about falling when walking in their neighborhood (16.54 %). The majority reported positive environmental perceptions of safety (73.16 %) and moderate or high neighborhood cohesion (64.71 %). Overall, the neighborhood median age measured at the Census Tract ranged from 22.8 to 43.3 with a median of 27.6. Table 1 also provides a descriptive view of how study characteristics differ by the two main walking outcomes. Table 1 further provides evidence of significant differences using Chi Square tests in the respondents characteristics between frequent walkers and non-frequent walkers, and between those who meet vs. fail to meet the Surgeon General’s recommendations for PA through walking.

**Table 1:** Study characteristics by walking levels for any purpose per week

		Total		Frequent walker versus non-frequent walker					Meeting the CDC recommended guidelines for physical activity versus not				
				Having at least 3 days of walking for any purpose		Not Having at least 3 days of walking for any purpose			Having at least 150 min of walking for any purpose		Not Having at least 150 min of walking for any purpose		
		n	Percent	n	Percent	n	Percent	*p*-value	n	Percent	n	Percent	*p*-value
Demographic													
Education	High school or less	65	23.99	31	11.52	33	12.27	0.8026	12	4.60	49	18.77	<.0001
	More than high school	206	76.01	127	47.21	78	29.00	0.0006	61	23.37	139	53.26	<.0001
Sex	Male	135	49.63	85	31.48	50	18.52	0.0026	38	14.50	94	35.88	<.0001
	Female	137	50.37	74	27.41	61	22.59	0.2632	35	13.36	95	36.26	<.0001
Age	60–69 years	142	52.21	79	29.26	63	23.33	0.1794	33	12.60	104	39.69	<.0001
	70+	130	47.79	80	29.63	48	17.78	0.0047	40	15.27	85	32.44	<.0001
Race	Minority	41	15.13	22	8.18	19	7.06	0.6394	11	4.21	26	9.96	0.0137
	White	230	84.87	136	50.56	92	34.20	0.0036	62	23.75	162	62.07	<.0001
Health													
Number of days of poor physical health (past 30 days)	At/above 2 days	71	26.20	42	15.56	28	10.37	0.0943	14	5.36	56	21.46	<.0001
	1 or fewer	200	73.80	117	43.33	83	30.74	0.0162	59	22.61	132	50.57	<.0001
Number of days of poor mental health (past 30 days)	At/above 2 days	72	26.47	29	10.74	42	15.56	0.1229	10	3.82	61	23.28	<.0001
	1 or fewer	200	73.53	130	48.15	69	25.56	<.0001	63	24.05	128	48.85	<.0001
Worried about falling when walking in neighborhood	Yes	45	16.54	20	7.41	25	9.26	0.4561	9	3.44	31	11.83	0.0005
	No	227	83.46	139	51.48	86	31.85	0.0004	64	24.43	158	60.31	<.0001
Social													
Marital status	Single	62	22.96	29	10.82	33	12.31	0.6115	10	3.85	47	18.08	<.0001
	Married	208	77.04	128	47.76	78	29.10	0.0005	63	24.23	140	53.85	<.0001
Social support for walking (dog &/or person to walk with)	Yes to none	84	30.88	39	14.44	44	16.30	0.5831	16	6.11	65	24.81	<.0001
	Yes to one	134	49.26	82	30.37	51	18.89	0.0072	36	13.74	92	35.11	<.0001
	Yes to both	54	19.85	38	14.07	16	5.93	0.0028	21	8.02	32	12.21	0.1308
Neighborhood cohesion	Low	96	35.29	45	16.67	50	18.52	0.6080	22	8.40	67	25.57	<.0001
	Moderate	104	38.24	59	21.85	44	16.30	0.1394	24	9.16	79	30.15	<.0001
	High	72	26.47	55	20.37	17	6.30	<.0001	27	10.31	43	16.41	0.0558
Median age (census tract)	At/Lower than the median	146	53.68	93	34.44	51	18.89	0.0005	45	17.18	91	34.73	<.0001
	Higher than the median	126	46.32	66	24.44	60	22.22	0.5930	28	10.69	98	37.40	<.0001
Positive environmental perceptions of safety	Low	30	11.03	18	6.67	12	4.44	0.2733	10	3.82	18	6.87	0.1306
	Moderate	43	15.81	25	9.26	18	6.67	0.2858	11	4.20	31	11.83	0.0020
	High	199	73.16	116	42.96	81	30.00	0.0126	52	19.85	140	53.44	<.0001

*N* = 272 with less than 5 % variation for missing data

Cell sizes and percentages reflect each control variable by the each of the two outcome variables of interest. For example, with education across the outcome meeting/not meeting the CDC recommended physical activity: 12 (4.60 %) + 49 (18.77 %) + 61 (23.37 %) + 139 (53.26 %) total 100 %. Bivariate logistic analyses (not shown) revealed that poor mental health, fears about falling, marital status, social support for walking, neighborhood cohesion, and median age (census tract-level) variables were significantly (*p* < .05) associated with frequent walking behaviors. Additionally, poor mental health days, marital status, social support for walking, and neighborhood cohesion variables were significantly (*p* < .05) associated with meeting CDC guidelines

### Walking patterns and places

**Fig. 2:**
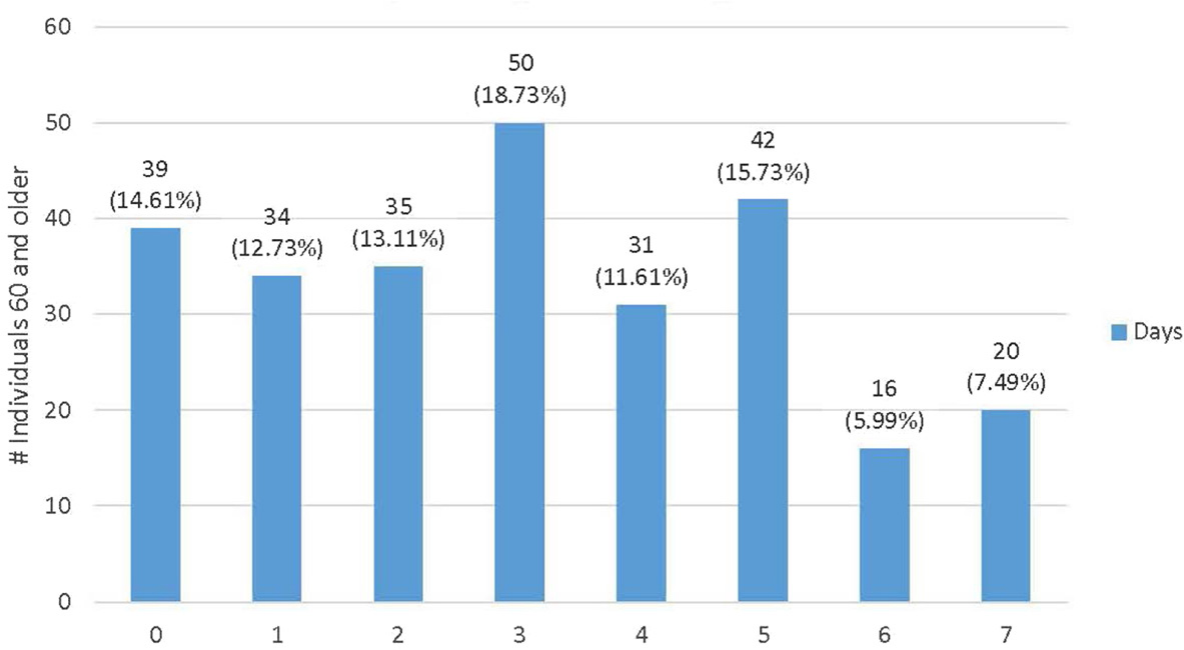
Days of walking in one’s neighborhood a typical week (*n* = 267)

**Fig. 3:**
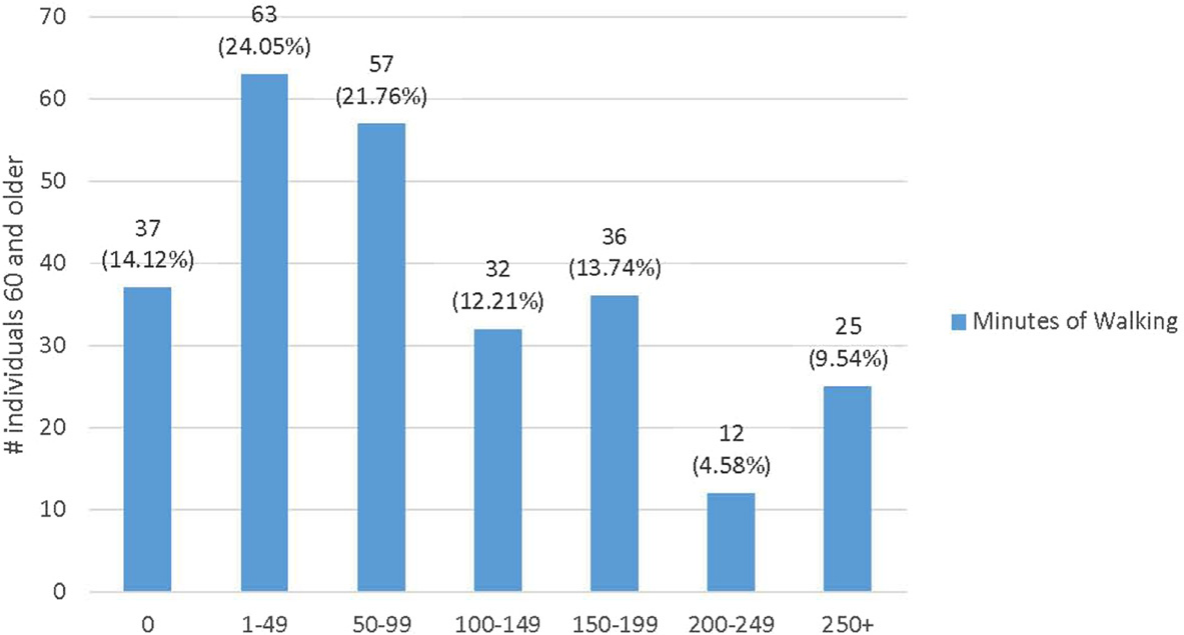
Total minutes of walking in one’s neighborhood in a typical week (*n* = 262)

As further indicated in Figs. 2 and 3, there was variability in walking behaviors. Overall, the median number of days individuals walked was 3, with the median number of minutes per week at 80 min. While the majority (59.55 %) walked at least three times a week, 14.61 % did not walk in their neighborhoods at least weekly (see Fig. 2). Further, only 27.86 % walked ≥150 min a week and 9.4 % walked 250 or more minutes per week (see Fig. 3).

Participants reported a variety of places where they walked in their neighborhood at least once a week. Overall, 75.38 % (196 out of 260 who answered this question) reported neighborhood streets, followed by green areas (*n* = 89, 34.23 %), malls (*n* = 67, 25.77 %), gyms (*n* = 19, 7.31 %), and schools (*n* = 15, 5.77 %). Other less frequently used places mentioned included treadmills at home, around large yards or properties, along the highway, or at golf courses (*n* = 31, 11.92 %). Since participants could indicate more than one place, percentages do not add up to 100 %.

### Multivariate analyses

Table 2 presents results of multivariate analyses. An examination of factors associated with frequent walking versus non-frequent walking behaviors reveals no significant demographic predictors. With regard to health-related variables, those reporting two or more poor mental health days (per month) are 65.5 % less likely to report frequent walking behaviors than those reporting fewer poor mental health days (OR = 0.345, 95 % CI = 0.185–0.645). Regarding environmental factors, those living in areas with younger population (census tract) median age are almost 80 % more likely to report frequent walking behaviors (OR = 1.799, 95 % CI = 1.034–3.131) than those in areas where the population is older. Compared to those reporting a high level of social cohesion, those reporting a low level are 66.2 % (OR = 0.338, 95 % CI = 0.159–0.715) less likely and those reporting a moderate level are 52.9 % (OR = 0.471, 95 % CI = 0.228–0.974) less likely to report frequent walking behaviors.

**Table 2:** Multiple logistic regression of demographic, health, social, and environmental factors associated with two walking behaviors among older adults

		Frequent versus non-frequent behavior			Meeting the CDC recommended guidelines for physical activity versus not		
		Having at least 3 days of walking for any purpose			Having at least 150 min of walking for any purpose		
		Odds Ratio	Lower 95 % CI	Upper 95 % CI	Odds Ratio	Lower 95 % CI	Upper 95 % CI
Demographic							
Education	High school or less versus more than high school	0.630	0.327	1.213	0.531	0.241	1.169
Sex	Male versus female	1.027	0.582	1.809	0.765	0.409	1.431
Age	60–69 years versus 70+	0.660	0.371	1.173	0.538^a^	0.290	0.997
Race	Minority versus White	0.963	0.444	2.089	1.684	0.699	4.057
Health							
Number of days of poor physical health (past 30 days)	At/above 2 days versus 1 or fewer	1.556	0.818	2.959	0.576	0.276	1.200
Number of days of poor mental health (past 30 days)	At/above 2 days versus 1 or fewer	0.345^a^	0.185	0.645	0.432^a^	0.198	0.944
Worried about falling when walking in neighborhood	Yes versus no	0.635	0.304	1.326	0.757	0.310	1.852
Social							
Marital status	Single versus married	0.772	0.392	1.521	0.684	0.292	1.598
Social support for walking (dog &/or person to walk with)	Yes to none versus yes to both	0.532	0.226	1.254	0.383^a^	0.154	0.957
	Yes to one versus yes to both	0.760	0.352	1.643	0.589	0.275	1.260
Neighborhood cohesion	Low versus high	0.338^a^	0.159	0.715	0.721	0.330	1.577
	Moderate versus high	0.471^a^	0.228	0.974	0.617	0.297	1.283
Median age (census tract)	At/Lower than the median versus higher	1.799^a^	1.034	3.131	1.737	0.950	3.175
Positive environmental perceptions of safety	Low versus high	1.296	0.516	3.253	2.172	0.820	5.756
	Moderate versus high	1.040	0.489	2.213	1.022	0.445	2.347

^a^Indicates significant using alpha at 0.05

An examination of factors associated with meeting versus not meeting the Surgeon General’s recommendations for PA through walking reveals that age is a significant predictor. Those who are younger (60–69 years) versus older (70 years or older) are 46.2 % less likely to meet these recommendations (OR = 0.538, 95 % CI = 0.290–0.997). Those reporting poorer mental health are 56.8 % less likely to meet these recommendations than those reporting fewer poor mental health days (OR 0.432, 95 % CI = 0.198-.944). Those reporting no human or animal companions to walk with are 61.7 % less likely to meet these recommendations than those who have both sources of social support for walking (OR = 0.383, 95 % CI = 0.154–0.957).

## Discussion

Individuals in this study experienced many previously reported facilitators to being physically active. They were in relatively good health [31, 32], expressed few concerns about falling [33], had social supports for motivating activity [30, 34], and lived in environments with good neighborhood cohesion [35, 36]. These facilitators may account for the majority of conditions that frequent walkers have. Yet, consistent with other national research [37], the majority of older adults were not meeting the Surgeon General’s PA recommendations. This calls for a better understanding of the intersection of social and environmental factors as predictors of walking among older adults.

This study confirms that a sizable amount of neighborhood walking by older adults is done in neighborhood environments, however, other research suggests that this may vary depending on the neighborhood characteristics and individuals’ mobility [38]. However, a significant number of respondents (>25 %) reported utilizing malls as a place for walking, providing support for mall walking as a safe and convenient venue for being physically active especially among older adults [39]. Given the relative homogeneity of the population, in terms of age, health status, and general socioeconomic status, it is not surprising that there were fewer significant associations than in other walking studies with significant findings for age [3] and neighborhood socioeconomic status [40].

Although there was some variability in poor physical health days, screening out those with more extreme difficulty in walking might have attenuated the impact of this variable shown in other studies [41]. The strong association with poor mental health days for both walking outcome variables reinforces other studies calling for greater attention to the effects of mental health on health behaviors [42] and general health outcomes [43, 44]. This is especially important given that emotional health concerns (including depression) are prevalent but typically underdiagnosed in older adults [43].

We are intrigued by the finding that having multiple sources of social engagement for walking (e.g., both human and companion animal supports) was predictive at least in terms of meeting the Surgeon General’s PA recommendation. This observation suggests the need for more research on the multiplicative effects of different sources of social support, since having just one source of support was not predictive of walking behaviors. Thirdly, in this study neighborhood cohesion emerged as a significant factor in contrast to some other studies [45, 46], but traffic concerns did not. Further research is needed to explore if those with traffic concerns are going to more protective environments, either inside to fitness centers, malls, or to parks/trails which may provide more conducive environments for being active.

While many similarities were found for factors differentiating non-frequent activity patterns from those associated with achieving recommended PA levels, there were some notable differences, confirming earlier literature suggesting that sedentary behavior is a distinct concept from more minute-based PA levels [47, 48]. For example, direct social support was associated with meeting PA guidelines, while neighborhood social cohesion and age composition appeared significant in encouraging more minimal levels of walking behaviors.

### Limitations

While we see significant contributions of this study in terms to adding to the knowledge base on the linkage between environmental factors and walking behaviors among older adults, there are several limitations which must be acknowledged.

While assessing electronic medical records provided an easy way to identify potential subjects, over 7000 patient records were needed to identify about 500 subjects. This low recruitment yield is unfortunately typical in survey-based studies in primary care settings with older adults [49, 50]. We are aware that we do not have a clinical population representative of our integrated health care system. Rather, given our screening criteria and limitation to individuals 60 years or older, our study population represents a subset of the larger population. The demographics of the study participants are described to frame the type of populations to which our findings may be most appropriately generalized. For example, participants were limited to four communities in central Texas and, as such, findings may not be generalized to other states or national populations with different socioeconomic characteristics.

Another limitation is the self-reported nature of the data, which may lead to misreporting of some of the variables. This is especially so for reporting walking behaviors, although we are more confident in the accuracy of reported days versus actual cumulative minutes. We acknowledge that our PA measures may be somewhat crude, and do not take into account that patients may be getting other types of exercise. Also, we did not specify walking at a moderate pace, so our reference to the Surgeon General’s recommendation must be considered approximate versus definitive. While our cut-point for non-frequent physically active behavior has been justified in previous literature [21–23], due to our sample distribution, we were not able to examine those reporting zero days of PA. Examining other PA measures in a larger and more diverse population may result in different associations.

Further, the data set was limited to a fixed set of questions limiting the ability to examine relationships among a full set of psychosocial predictors or outcomes of interest (e.g., we did not have questions assessing patient motivations or other sedentary behaviors such as sitting or screen time). Further, we did not account for variation in employment status, which may influence one’s PA. In addition, we were not able to report on the relationship between walking and all potential neighborhood factors that may influence walking. For example, danger from others such as gun violence or mugging may have influenced our outcomes but was not measured in the current study. Finally, our data are cross-sectional and do not permit an understanding of the causal chains linking the different variables of interest.

### Implications for research, practice, and policy

Nevertheless, the current study adds to the literature on how place matters with regard to different aspects of PA among older adults and suggests further research, practice and policy action. Several factors deserve more attention. We note that the aggregate population age of one’s surrounding can act as a facilitator of PA, with older adults living in areas more populated by younger adults being more likely to be physically active. It is not clear whether older adults are influenced by seeing more active neighbors, or if neighborhoods catering to younger populations have more amenities promoting PA. This finding though supports the 8–80 philosophy of having activity friendly environments where persons of all ages can safely be active, and calls for more research into intergenerational influences on PA [32].

Further, identifying facilitators of PA in one’s environment provides guidance for interventions at the environmental level. Future analyses including objective measures of both the physical place and walking behaviors among older adults can help elucidate relationships between health and place. In future studies, we recommend longitudinal analyses to enable assessments of dynamic changes over time. In particular, we would want to test the interplay among the social and built environments, lifestyle factors, and health outcomes [40] to better understand the complex interactions among sociodemographic, health, environmental, and walking behaviors.

Additional longitudinal research with larger and more diverse populations should be conducted to identify how different aspects of the environment (e.g., natural, built or social) interact with personal factors to encourage PA and maximize population health for older adults [3]. This includes greater attention to the need for targeted research funding and an appreciation of the complexities of this type of environmental research.

In recognition of the health benefits of PA, we endorse clinical and sports medicine professional organizations dissemination of “exercise as medicine” messages to help Americans live healthier lives [51]. In line with the “exercise as medicine” philosophy, we encourage physicians to assess and advise their older patients on how they can be more physically active for health promotion and disease prevention. Additionally, we endorse efforts underway to inform physicians of the impact of environmental factors on their patient’s health [52] and to create and disseminate inventories of places where older adults can safely walk [53, 54]. Recognizing the important influence that mental health concerns can have on initiating and sustaining healthy lifestyles [37], consistent with clinical guidelines for older patients [55], we also recommend that physicians assess whether late-life depression or other mental health symptomatology are present and how these can be addressed to enhance activity levels and overall quality of life.

## Conclusion

Physicians are critical change agents for motivating positive lifestyle behaviors [11]. However, we stress the importance of all stakeholders working together to create more active friendly environments that can impact greater population health [56]. Nationally, the U.S. Surgeon General’s Office has embarked on a new “America Walks” campaign [30] to get Americans walking more through a coalition of local, state, and national advocacy groups that emphasize the importance of changing social norms and providing social supports so that being active will be “the new normal” for Americans of all ages. This complements the 8–80 cities movement where even small changes in the environment such as “open streets” can have tremendous impacts on communities helping both young and old meet PA recommendations [57]. Further, we recommend that urban planners stress the importance of age-integrated communities for promoting PA for all and consider design factors that enhance neighborhood cohesion [52]. These multifaceted activities will enable researchers, practitioners, and policy makers to work together to provide the critical building blocks for aging in place.

## Abbreviations

PA: Physical activity
CDC: Centers for Disease Control And Prevention
OR: Odds ratio
BRFSS: Behavioral Risk Factor Surveillance System
NEWS: Neighborhood Environment Walkability Scale
IRB: Institutional Review Board
